# A Phenomenological Primary–Secondary–Tertiary Creep Model for Polymer-Bonded Composite Materials

**DOI:** 10.3390/polym13142353

**Published:** 2021-07-18

**Authors:** Xiaochang Duan, Hongwei Yuan, Wei Tang, Jingjing He, Xuefei Guan

**Affiliations:** 1 Graduate School of China Academy of Engineering Physics, Beijing 100193, China; duanxiaochang19@gscaep.ac.cn; 2 Institute of Chemical Materials, China Academy of Engineering Physics, Mianyang 621900, China; yuanhw@caep.cn (H.Y.); tangwei@caep.cn (W.T.); 3 School of Reliability and Systems Engineering, Beihang University, Beijing 100191, China; hejingjing@buaa.edu.cn

**Keywords:** polymer-bonded composites material, primary–secondary–tertiary creep, temperature- and stress-dependent, phenomenological model

## Abstract

This study develops a unified phenomenological creep model for polymer-bonded composite materials, allowing for predicting the creep behavior in the three creep stages, namely the primary, the secondary, and the tertiary stages under sustained compressive stresses. Creep testing is performed using material specimens under several conditions with a temperature range of 20 °C–50 °C and a compressive stress range of 15 MPa–25 MPa. The testing data reveal that the strain rate–time response exhibits the transient, steady, and unstable stages under each of the testing conditions. A rational function-based creep rate equation is proposed to describe the full creep behavior under each of the testing conditions. By further correlating the resulting model parameters with temperature and stress and developing a Larson–Miller parameter-based rupture time prediction model, a unified phenomenological model is established. An independent validation dataset and third-party testing data are used to verify the effectiveness and accuracy of the proposed model. The performance of the proposed model is compared with that of an existing reference model. The verification and comparison results show that the model can describe all the three stages of the creep process, and the proposed model outperforms the reference model by yielding 28.5% smaller root mean squared errors on average.

## 1. Introduction

Creep is a time-dependent progressive inelastic deformation behavior. It can cause the relaxation of stress and irreversible deformation, thus leading to functional failures when the part is intended to maintain the required stress and shape [1]. Polymer-bonded composites materials (PBMs) are increasingly used in engineering components due to its high strength and lightweight [2,3,4]. For PBMs the creep can take place at a relatively low temperature [5]; therefore, a reliable prediction of the creep behavior is critical to ensure the safety and durability of PBMs under sustained loads.

**Figure 1:**
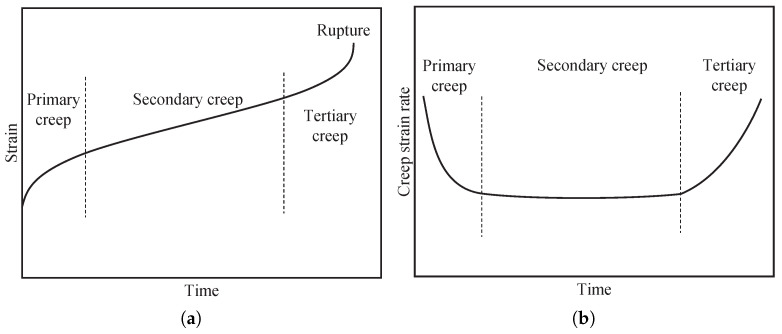
Creep response of typical PBMs. (**a**) The complete creep process curve, and (**b**) the creep strain rate vs. time curve.

Several studies have shown that the creep behavior of PBMs depends on many factors such as matrix content [6,7,8,9], particle size [10,11,12], stress [13,14,15], temperature [16,17], and humidity [18,19]. The importance of long-term properties for polymer composites are highlighted in recent studies [20,21]. Experimental results reveal that the entire process of creep deformation can be divided into three stages [22,23], namely the primary (transient) creep, the secondary (stationary) creep, and the tertiary (unstable) creep. The three stages of the creep process are illustrated in Figure 1a with the corresponding creep strain rate shown in Figure 1b. The secondary (stationary) creep is considered to be the dominant creep for many applications. In this stage, the equilibrium between the softening and hardening of the material is assumed, leading to a stable strain rate [24,25,26]. Prior to the stationary stage, a short transient period of primary creep is required to reach such an approximate equilibrium between the softening and hardening processes. The final part of the creep process is the tertiary stage where the strain rate increases rapidly until rupture [27,28]. The progressive damage such as the formation and growth of voids on grain boundaries are considered to be the main contributors of the rapid growth of the strain rate.

Traditionally the three stages are modeled separately. For primary creep, the creep rate equations are in general given by [29]

(1) $$\dot{\varepsilon }=\frac{d\varepsilon }{dt}=\left\{\begin{array}{cc} a\sigma^{n}\cdot t^{m} & \mathrm{Time hardening}\\ a\sigma^{n}\cdot \varepsilon^{m} & \mathrm{Strain hardening} \end{array}\right.,$$

where *a*, *n*, and *m* are fitting parameters. For the secondary creep, the equivalent creep rate equations are given by Equation (2) [30,31,32,33,34,35,36],

(2) $$\dot{\varepsilon }=\frac{d\varepsilon }{dt}=\left\{\begin{array}{cc} a\sigma^{n} & \mathrm{Norton, Bailey}\\ b\left(\exp\frac{\sigma }{\sigma_{0}}-1\right) & \mathrm{Soderberg}\\ a\sinh\frac{\sigma }{\sigma_{0}} & \mathrm{Prandtl, Nadai}\\ a_{1}\sigma^{n_{1}}+a_{2}\sigma^{n_{2}} & \mathrm{Johnson et al.}\\ a{\left(\sinh\frac{\sigma }{\sigma_{0}}\right)}^{n} & \mathrm{Garofalo} \end{array}\right.,$$

where *a*, *b*, $a_{1}$, $a_{2}$, $\sigma_{0}$, *n*, $n_{1}$, and $n_{2}$ are fitting parameters, and σ is the effective stress, e.g., the equivalent stress. To accommodate the temperature effect, the Arrhenius law is usually incorporated into Equations (1) and (2).

Existing constitutive modeling methods for the creep behavior under different testing conditions can be classified into three categories, the general stress–strain–time modeling, the rheological modeling, and the empirical modeling [37]. The generalized stress–strain–time modeling is used to describe the viscous effect and rate-independent behavior under general loading conditions, including the creep model based on the elastic–viscoelastic correspondence principle and a material stiffness equation [38], the Wiechert model with damage evolution law and time–temperature shift factor for creep response prediction under different temperature [39], the micromechanics model employing the correspondence principle in viscoelasticity [40], and the viscoelasticity–viscoplasticity temperature-dependent model that includes anisotropic damage evolution [41]. The rheological modeling is centered on the idea of using basic components such as springs, dashpots, and sliders to describe the creep strain behavior with time. The creep model of PBM can be formulated by combining various basic components with the viscoelastic principle. For example, the Burgers model based on time–temperature equivalence principle [28,42], the fractional Poynting-Thomson model [43], and the viscoelastic response-based models [44,45]. The empirical modeling is to establish the correlations between the creep rate and other independent variables such as temperature and stress using testing data. Examples of this type of model include, but are not limited to, the stress-dependent phenomenological viscoelastic–plastic model [46], the modified power law model with temperature- and stress-dependent correction factors [47], and the improved Findley–Khosla model with the Schapery’s integral [48]. For empirical modeling, the detailed mechanisms of the creep behavior are not fully explained. However, when the boundary conditions are consistent with the experiment, it can provide solutions for practical engineering problems [49].

The models for predicting the full-stage creep behavior of PBMs are limited. Krankel et al. developed a rheological model by introducing the time variable into the Burgers model for creep behavior prediction of bonded anchors [50]. Sudduth et al. developed a polynomial strain rate model which enables the capture of the behavior of the full creep curve [51]. However, the above two models cannot directly incorporate the dependence of model parameters on temperature and holding stress, making it difficult to predict the creep strain under more general conditions without testing data. The purpose of this study is to develop a unified phenomenological creep model for polymer-bonded composite materials, allowing for prediction of the creep behavior and life under more general conditions without testing data. To achieve that, the temperature- and stress-dependent effect is incorporated in the proposed rational function-based full creep strain rate equation, and the creep rupture time model based on the Larson–Miller parameter is developed. The former creep rate equation differs from the existing full-stage creep rate models in format and uses less (three) fitting parameters. The latter rupture time model under general conditions without testing data is rarely seen for PBMs.

The remainder of the study is organized as follows. First, the experimental work and creep data of a typical PBM are presented. Uni-axial compression creep testing in the temperature range of 20 °C–50 °C and the holding stress range of 15 MPa–25 MPa is performed to obtain the full-stage creep data. Next, a rational function-based phenomenological model is proposed to describe the creep behavior in the entire primary–secondary–tertiary process. By correlating the fitting parameters with temperature and stress, a general temperature- and stress-dependent full-stage creep model is formulated. The creep rupture time model is developed using the Larson–Miller parameter. Following that, the model is validated using independent testing data and third-party testing data of another type of PBM. The performance of the proposed model is further compared with an existing reference model. Finally, conclusions are drawn based on current results.

## 2. Experimental Testing

**Figure 2:**
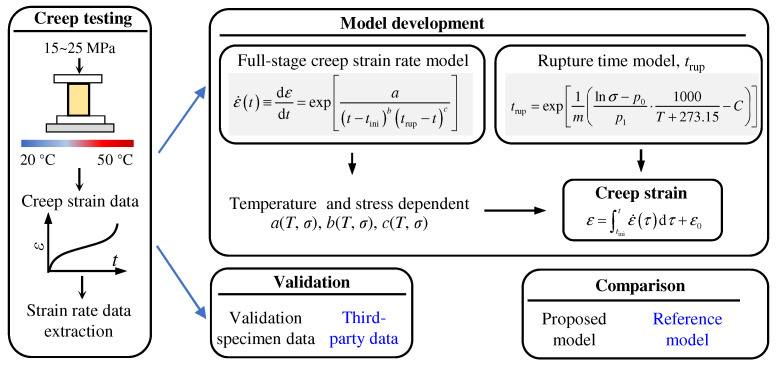
Overall diagram of creep testing, model development, validation, and comparison.

Figure 2 presents the procedure of the overall methodology development of this study. In the experimental part, a total number of six specimens are prepared for uni-axial compression creep testing at temperatures ranging from 20 °C to 50 °C with a holding stress of 15 MPa–25 MPa. The total strain vs. time data are acquired for each of the specimens, and the strain rate data are extracted using a central difference scheme for strain rate model development.

### 2.1. Specimens and Experimental Setup

**Figure 3:**
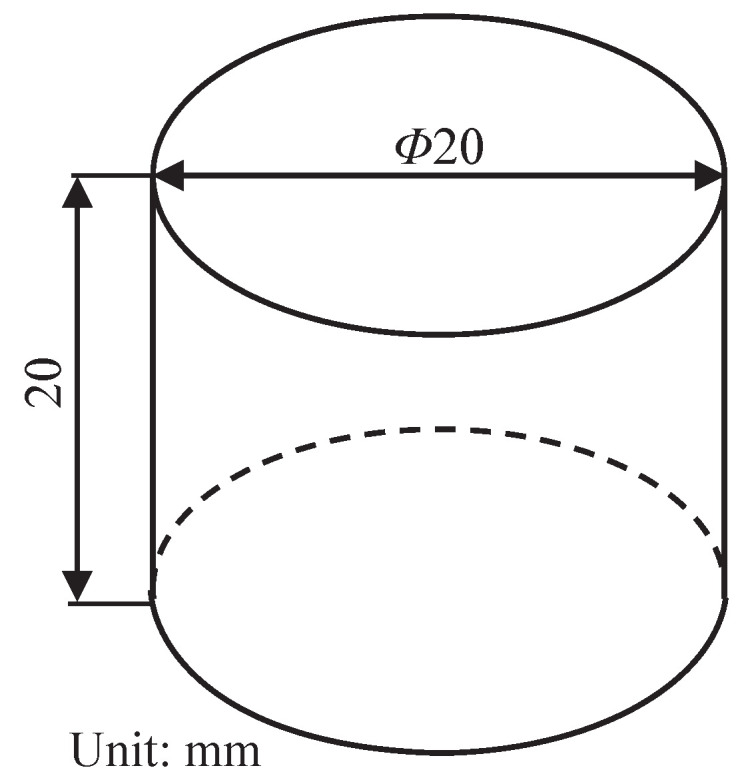
Geometry and dimension of the specimen.

The PBM used in this study consists of 94 wt.% barium sulfate grains as filler material and 6 wt.% fluororubber as matrix material. The size of the filler is in the range of 0.5 mm and 3 mm. Cylinder specimens with dimensions shown in Figure 3 are prepared according to the standard [52].

**Figure 4:**
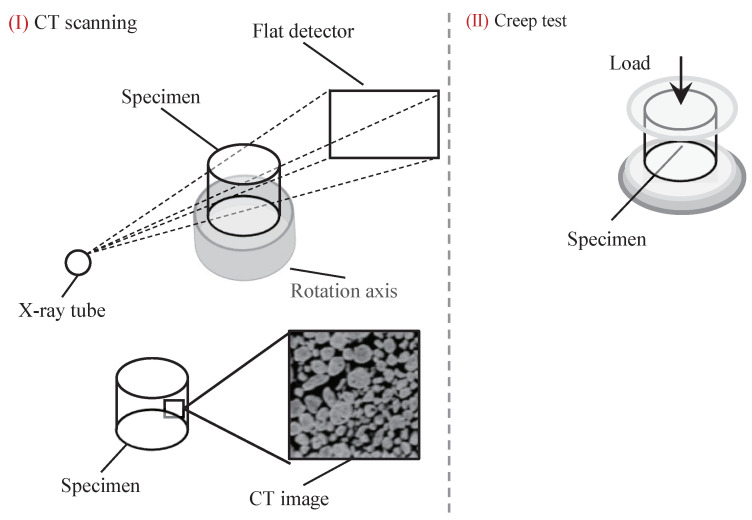
Schematic diagram of damage inspection process.

Prior to creep testing the specimens are examined using cone-beam computed tomography (CT) to ensure no initial damage exists in the materials. The inspection process is illustrated in in Figure 4, and a typical CT image is given where the light-colored filler particles are barium sulfate, and the rest dark area is the fluororubber binder material. A total number of six specimens are prepared, and testing conditions for the specimens are shown in Table 1. The uni-axial compression creep testing is performed using a universal testing machine with an environmental chamber. The environmental chamber allows for keeping the temperature at a prescribed value during the creep testing. The compressive stress is applied to the specimen at a strain rate of 0.5 mm/min until the prescribed stress is reached. After that the applied stress is sustained until rupture.

### 2.2. Creep Testing Results

**Figure 5:**
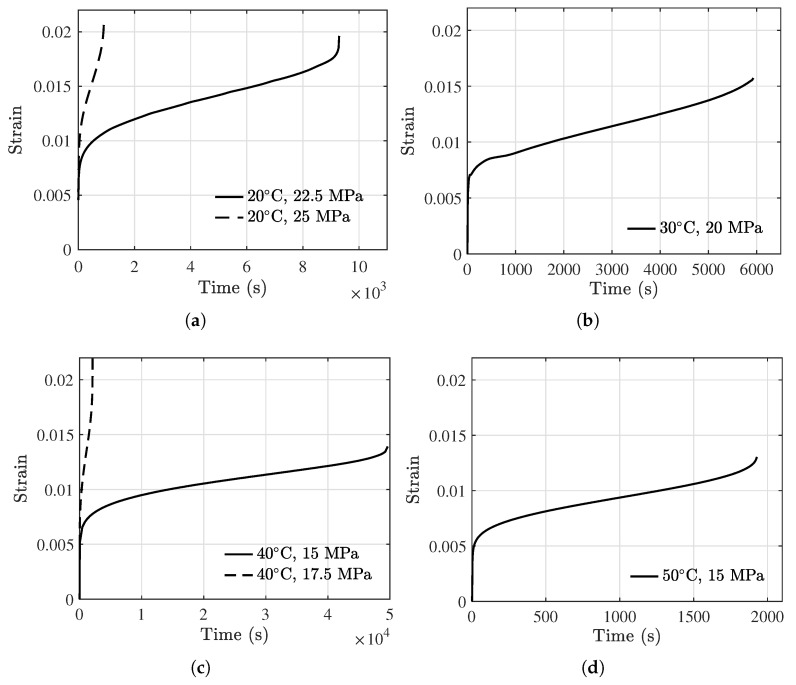
Creep testing results under different temperatures and stresses. (**a**) 20 °C, (**b**) 30 °C, (**c**) 40 °C, and (**d**) 50 °C.

The strain vs. time data of the six specimens from the initial state to rupture are acquired, and results are presented in Figure 5. It can be observed from Figure 5 that the creep behavior of the PBM exhibits three distinct stages. The initial rapidly increasing of the strain is mainly due to elastic deformation, plastic deformation, and work hardening. After that, the strain curve remains a relatively constant slope for a significant amount of time. Following that is another rapidly increasing of the strain leading to the final rupture. In addition, a higher holding stress can greatly reduce the rupture life as shown in Figure 5a,c. For example, the rupture time decreases from 46,900 s at 40 °C, 15 MPa to 1918 s at 50 °C, 15 MPa, and from 9292 s at 20 °C, 22.5 MPa to 916 s at 20 °C, 25 MPa.

### 2.3. Strain Rate Extraction

**Figure 6:**
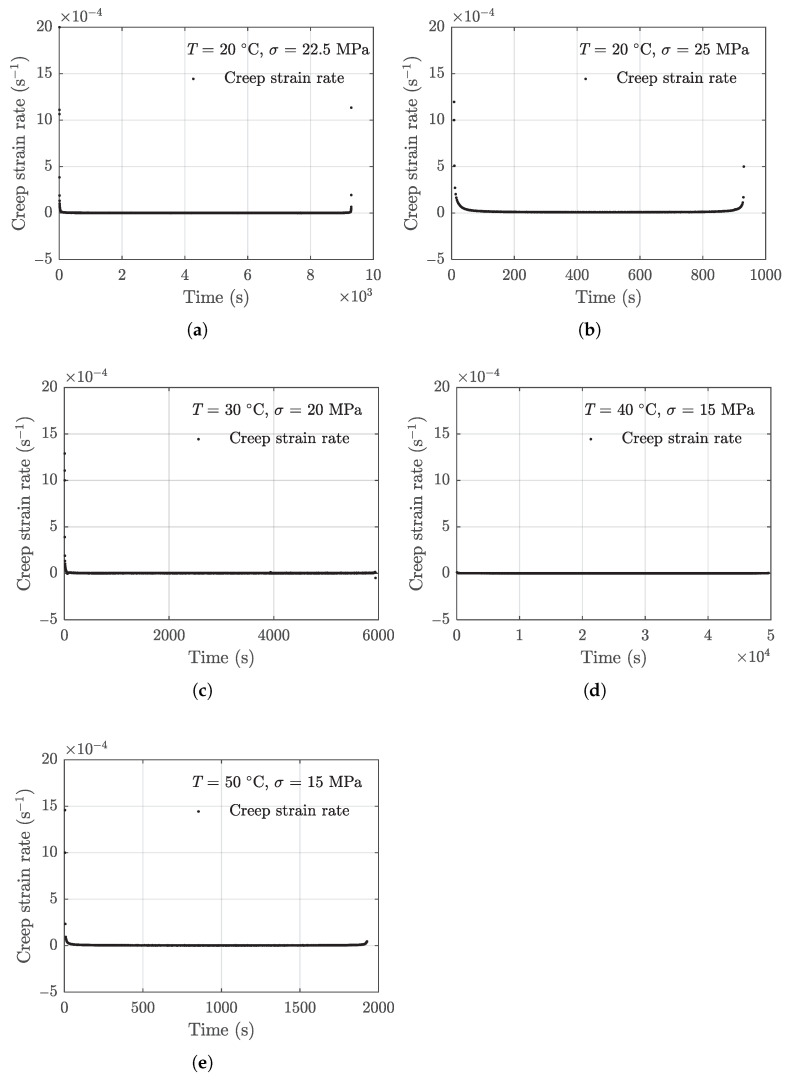
Creep strain rate data. (**a**) 20 °C, 22.5 MPa, (**b**) 20 °C, 25 MPa, (**c**) 30 °C, 20 MPa, (**d**) 40 °C, 15 MPa, (**e**) 50 °C, 15 MPa.

A central difference scheme given as Equations (3) and (4) is used to extract the strain rate from the strain vs. time data.

(3) $$\frac{d\varepsilon }{dt}=\frac{\varepsilon_{i+1}-\varepsilon_{i}}{t_{i+1}-t_{i}},i=1,2,3,\ldots ,n-1,$$

(4) $$t=\frac{t_{i+1}+t_{i}}{2},i=1,2,3,\ldots ,n-1,$$

where dε/dt is creep strain rate and the *t* is the time variable. The subscript *i* is the data point index and the total number of data points is *n*. The strain vs. time testing data are processed using Equations (3) and (4) to obtain the strain rate data. The extracted creep strain rate data of the five specimens used for model development are shown in Figure 6.

The results presented in Figure 6 show that the primary and tertiary stages are much shorter than the secondary stages, and creep rate in the entire creep process varies by up to several order of magnitudes. Therefore, direct modeling of the creep strain rate data in linear scale may yield undesired rounding errors due to such large differences in data. On the other hand, the strain variation is monotonic which ensures that the strain rate is positive, allowing for logarithm transformation. In this study, the log-transformed strain rate data are used for model development.

## 3. Creep Model Development

The log-transformed creep strain rate data under each of the testing conditions resemble a bathtub shape; therefore, a rational function–based equation is proposed to model the log-transformed strain rate data. The resulting fitting parameters of the model are subsequently correlate with temperature and stress using a response surface model to incorporate the effects of temperature and stress.

### 3.1. Creep Strain Rate Model

The following rational function–based equation is proposed to describe the log-transformed strain rate data.

(5) $$\ln\frac{d\varepsilon }{dt}=\frac{a}{{\left(t-t_{\mathrm{ini}}\right)}^{b}\cdot {\left(t_{\mathrm{rup}}-t\right)}^{c}},$$

where dε/dt and *t* are defined as before, and *a*, *b*, and *c* are the fitting parameters. The parameter *a* loosely measures the magnitude of the strain rate in the secondary stage, and the terms *b* and *c* are related to the transitions from the primary to the secondary and the secondary to the tertiary, respectively. The term $t_{\mathrm{ini}}$ is the initial time of the creep process, and $t_{\mathrm{rup}}$ is the creep rupture time.

**Figure 7:**
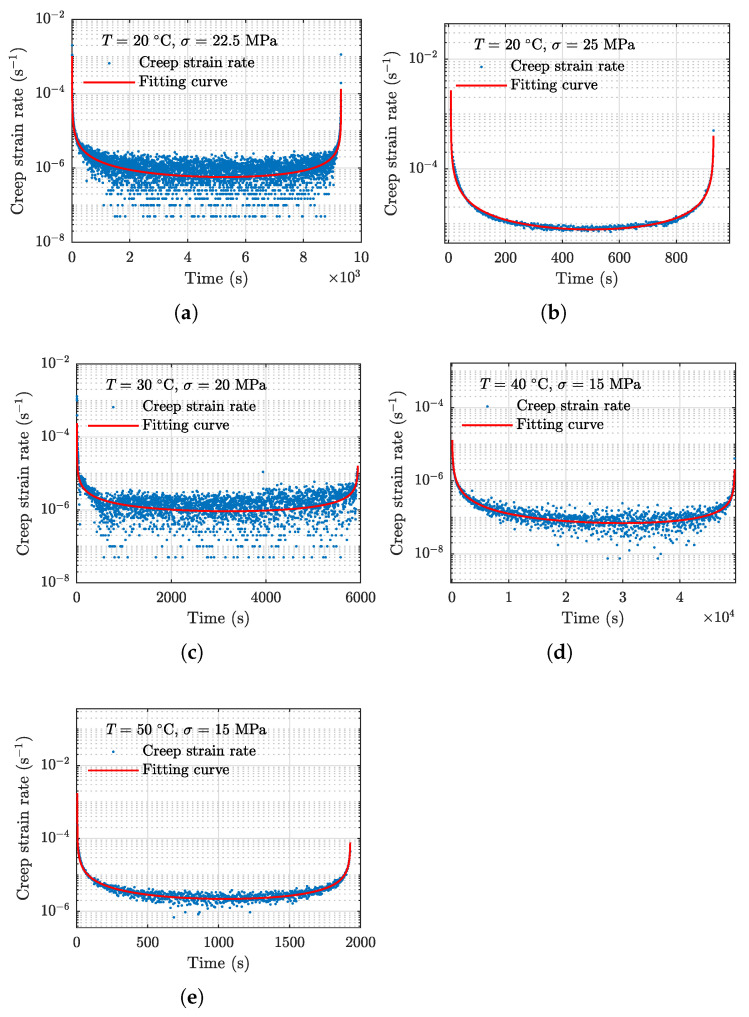
Results of mean curves obtained using Equation (5). (**a**) 20 °C, 22.5 MPa, (**b**) 20 °C, 25 MPa, (**c**) 30 °C, 20 MPa, (**d**) 40 °C, 15 MPa, and (**e**) 50 °C, 15 MPa.

The fitting parameters using data associated with the five specimens are obtained using the regular nonlinear least square estimator, and the resulting model parameters are presented in Table 2. With the fitting parameters, the mean curves of the proposed equation are computed and shown in Figure 7. It can be observed that the proposed creep strain rate equation Equation (5) can reliably capture the three stages of the actual strain rate data.

Exponentiation of Equation (5) to recover the strain rate in linear scale as

(6) $$\dot{\varepsilon }\left(t\right)\equiv \frac{d\varepsilon }{dt}=\exp\left[\frac{a}{{\left(t-t_{\mathrm{ini}}\right)}^{b}\cdot {\left(t_{\mathrm{rup}}-t\right)}^{c}}\right].$$

**Figure 8:**
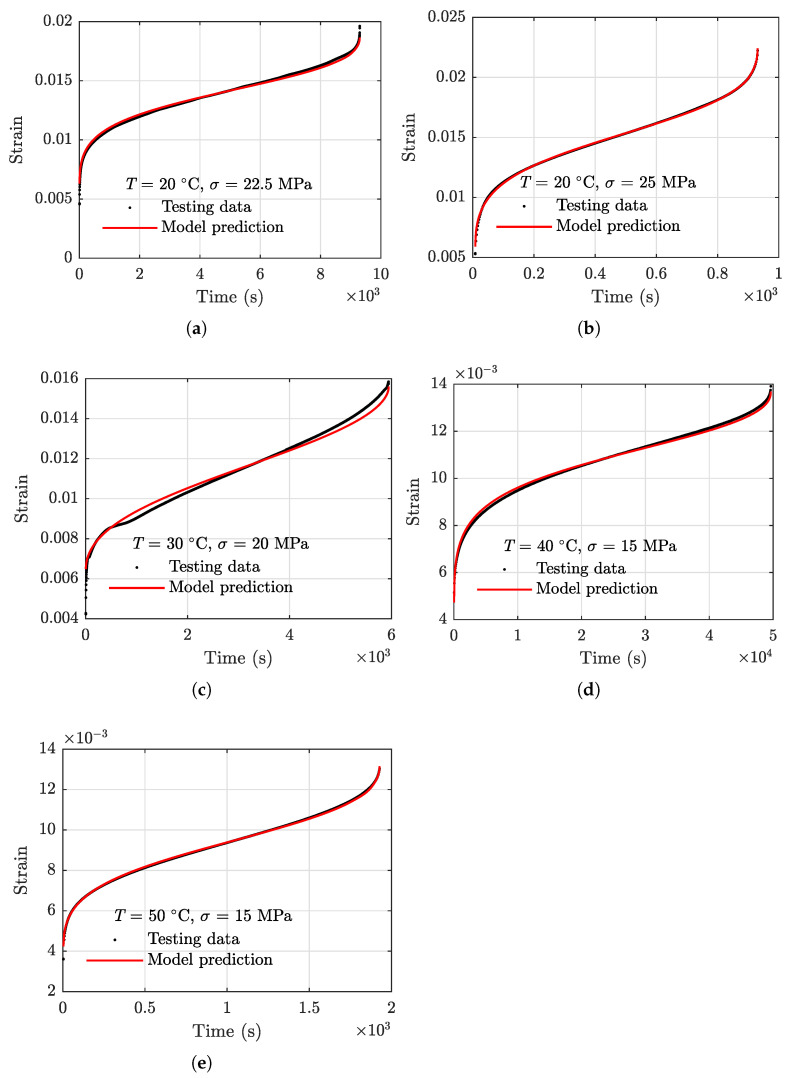
Results of creep strain prediction using Equation (7) with parameters in Table 2. (**a**) 20 °C, 22.5 MPa, (**b**) 20 °C, 25 MPa, (**c**) 30 °C, 20 MPa, (**d**) 40 °C, 15 MPa, and (**e**) 50 °C, 15 MPa.

The creep strain at a given time *t* can be obtained by time integration of Equation (6) from the initial time $t_{\mathrm{ini}}$ to *t* as

(7) $$\varepsilon \left(t\right)=\int_{t_{\mathrm{ini}}}^{t}\dot{\varepsilon }\left(\tau \right)d\tau +\varepsilon_{0},$$

where $\varepsilon_{0}$ is the transient elastic–plastic strain which can be set as a prescribed value in testing or estimated using stress–strain constitutive models [53]. It is noted that Equation (7) can be resolved using numerical integrators such as RK45 and its variants with the initial value of $\varepsilon_{0}$. Using Equation (7) and model parameters in Table 2, the strain vs. time results are obtained for the five specimens. Model results and the actual raw creep strain data are presented in Figure 8, where a close agreement between the two is observed.

### 3.2. Temperature and Stress Dependence Model

Based on the resulting model parameters under each of the conditions shown in Table 2, a first-order response surface model is employed to correlate the parameters with temperature and stress. The response surface model can be expressed as Equation (8).

(8) $$\left\{\begin{array}{c} a=\alpha_{1}\cdot T+\alpha_{2}\cdot \sigma +\alpha_{3}\\ b=\beta_{1}\cdot T+\beta_{2}\cdot \sigma +\beta_{3}\\ c=\gamma_{1}\cdot T+\gamma_{2}\cdot \sigma +\gamma_{3} \end{array}\right.,$$

where the $\alpha_{i}$, $\beta_{i}$, and $\gamma_{i}$ (i=1,2,3) are fitting coefficients, *T* is the temperature, and σ is the stress.

Using the data in Table 2, the fitting coefficients $\alpha_{i}$, $\beta_{i}$, and $\gamma_{i},i=1,2,3$ are obtained using the regular least square estimator as

(9) $$\left\{\begin{array}{cc} \alpha =\;\left[\alpha_{1},\alpha_{2},\alpha_{3}\right] & =\;\left[0.06181,0.2518,-12.94\right]\\ \beta =\;\left[\beta_{1},\beta_{2},\beta_{3}\right] & =\;\left[-9.499\times 10^{-4},-0.003540,0.03993\right]\\ \gamma =\;\left[\gamma_{1},\gamma_{2},\gamma_{3}\right] & =\;\left[-0.001765,-0.006215,0.1298\right] \end{array}\right..$$

**Figure 9:**
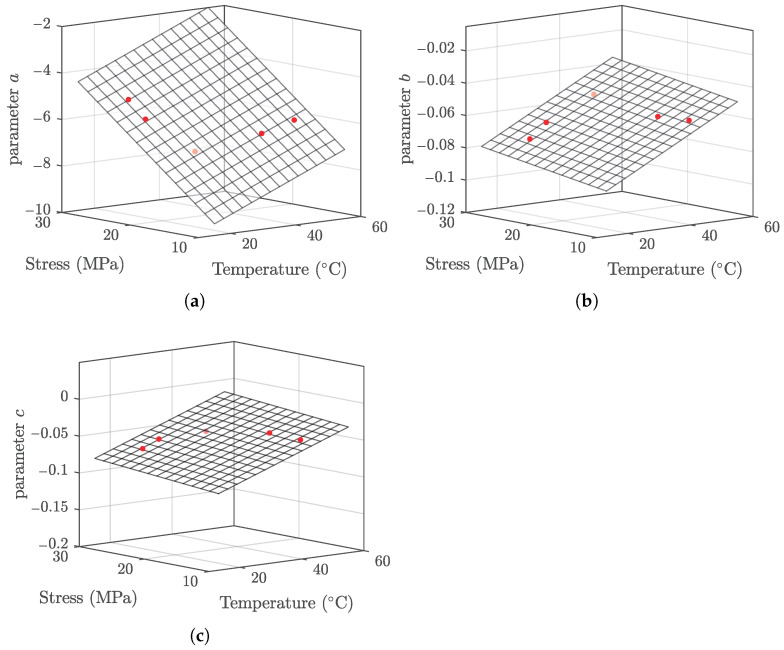
Temperature and stress dependence of parameters. (**a**) Parameter *a*, (**b**) parameter *b*, and (**c**) parameter *c*.

The prediction results of the response surface model with the fitting coefficients given in Equation (9) are evaluated and presented in Figure 9.

Combining Equation (5) and Equation (8) with the parameters in Equation (9), the temperature- and stress-dependent creep strain rate model can be expressed as

(10) $$\dot{\varepsilon }\left(t\right)\equiv \frac{d\varepsilon }{dt}=\exp\left[\frac{a(T,\sigma )}{{\left(t-t_{\mathrm{ini}}\right)}^{b(T,\sigma )}\cdot {\left(t_{\mathrm{rup}}-t\right)}^{c(T,\sigma )}}\right],$$

**Figure 10:**
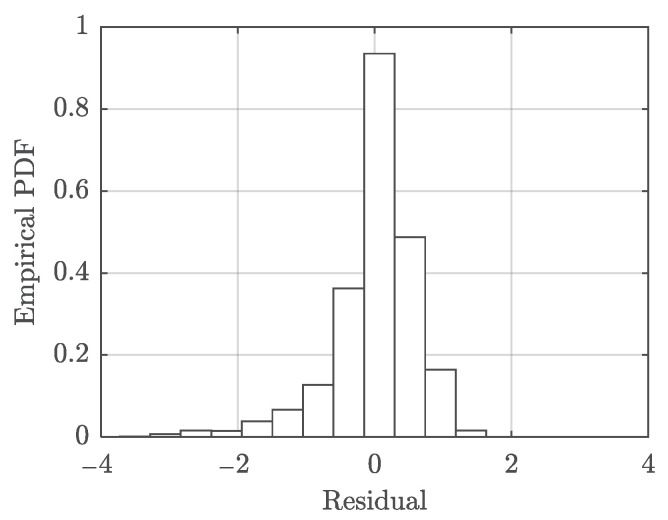
Model prediction residuals (in logarithm) of the developed strain rate model Equation (10).

The histogram of the model residuals is presented in Figure 10. The standard deviation of the residuals is estimated as 0.6382.

### 3.3. Creep Rupture Time Model

It is noted that the rupture time variable $t_{\mathrm{rup}}$ is required in the developed strain rate model Equation (10). The rupture time is defined as the time duration between the time when the part is loaded with the sustained stress and the time of the final fracture. In Equation (10), the rupture time can alter the tail region behavior of the strain curve. For conditions without tested specimens the corresponding rupture time is unknown. Consequently, the existing data on specimens tested under uni-axial compression are not sufficient for more general loading conditions. To predict the strain rate response under other conditions, it is necessary to establish rupture time prediction model to obtain $t_{\mathrm{rup}}$ for a given stress and a temperature.

**Figure 11:**
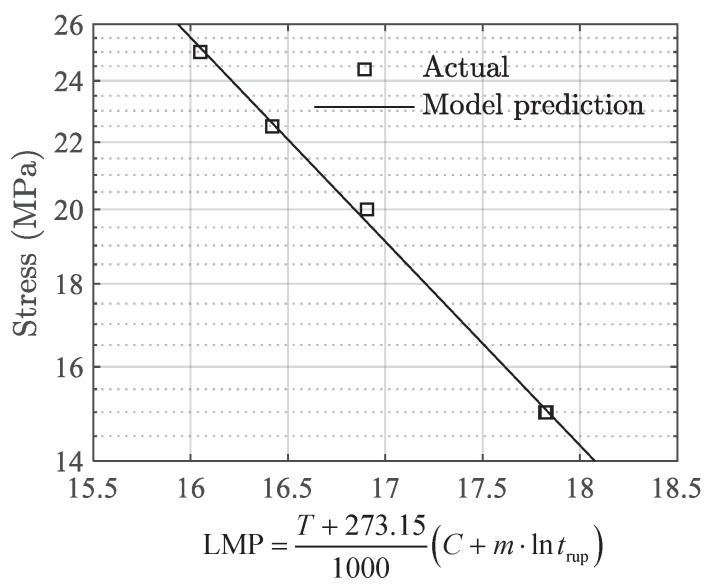
The actual and calculated results on stress vs. LMP.

In this study, the Larson–Miller parameter (LMP) [54] is adopted to develop the rupture time prediction model of PBMs. LMP is an equation to calculate the creep rupture time at different temperatures under a given stress. The basic form of the LMP can be expressed as

(11) $$\mathrm{LMP}=\frac{T+273.15}{1000}\left(C+m\cdot \ln t_{\mathrm{rup}}\right),$$

where *T* is the temperature in Celsius, $t_{\mathrm{rup}}$ is the creep rupture time, and *C* and *m* are material constants, respectively [54]. To further introduce the stress variable into the rupture time prediction, a linear relationship between the stress and the LMP is proposed as

(12) $$\ln\sigma =p_{0}+p_{1}\cdot \mathrm{LMP},$$

where $p_{0}$ and $p_{1}$ are fitting parameters. Using the rupture time, temperature, and stress data in Table 2, the optimal parameters of (C,m) are identified as (50.998,0.549) using the nonlinear least square estimator, and parameters $p_{0}$ and $p_{1}$ in Equation (12) are identified as (7.864,−0.289). With those parameters, the actual and model predicted results on stress vs. LMP are shown in Figure 11.

The rupture time under for a given combination of temperature and stress is obtained by substituting Equation (11) into Equation (12) as

(13) $$t_{\mathrm{rup}}\left(T,\sigma \right)=\exp\left[\frac{1}{m}\left(\frac{\ln\sigma -p_{0}}{p_{1}}\cdot \frac{1000}{T+273.15}-C\right)\right].$$

Incorporating Equation (13) into Equation (10) to obtain the final creep strain rate model as,

(14) $$\dot{\varepsilon }\left(t\right)\equiv \frac{d\varepsilon }{dt}=\exp\left[\frac{a(T,\sigma )}{{\left(t-t_{\mathrm{ini}}\right)}^{b(T,\sigma )}\cdot {\left[t_{\mathrm{rup}}\left(T,\sigma \right)-t\right]}^{c(T,\sigma )}}\right],$$

By further substituting Equation (14) into Equation (7) to have the final creep strain model

(15) $$\varepsilon \left(t\right)=\int_{t_{\mathrm{ini}}}^{t}\exp\left[\frac{a(T,\sigma )}{{\left(\tau -t_{\mathrm{ini}}\right)}^{b(T,\sigma )}\cdot {\left[t_{\mathrm{rup}}\left(T,\sigma \right)-\tau \right]}^{c(T,\sigma )}}\right]d\tau +\varepsilon_{0}.$$

**Figure 12:**
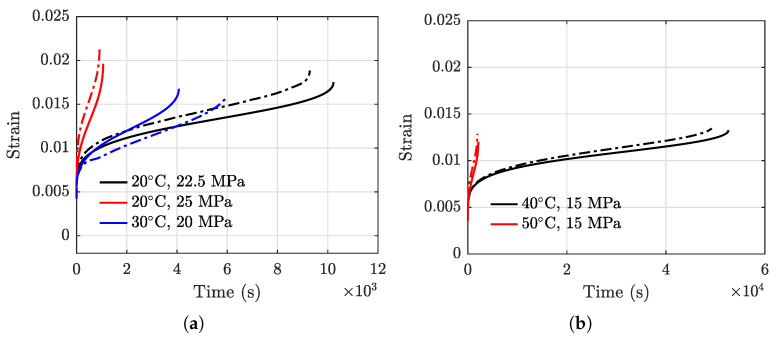
Comparisons of the predicted creep strain results (in solid lines) with the actual creep data (in dashed lines). (**a**) 20 °C, 30 °C, and (**b**) 40 °C, 50 °C.

The model prediction results using Equation (15) and the actual creep strain testing data are compared in Figure 12. To quantify the performance of the model, the root mean squared error (RMSE) defined in the following equation is employed.

(16) $$\mathrm{RMSE}=\sqrt{\frac{\sum_{i=1}^{N}{\left(y_{i}-{\hat{y}}_{i}\right)}^{2}}{N}},$$

where $y_{i}$ is the actual value, ${\hat{y}}_{i}$ is the prediction value, and i=1,⋯,N represents the index of a total number of *N* data points.

RMSEs of the testing data on the five specimens used for model development are calculated using Equation (14) and presented in Table 3. The maximum RMSE is 0.0027 for the testing data obtained under the condition of T=30 °C and σ=20 MPa.

## 4. Model Validations and Comparisons

An independent dataset, Specimen 5 in Table 1, is used to validate the performance of the model. Moreover, the third-party testing data reported in Ref. [55] are used to validate the effectiveness of the model for other PBMs. In addition, the proposed model is compared with an existing reference model to demonstrate its performance, and the performances of the two models in terms of RMSE are compared and quantified.

### 4.1. Model Validation

**Figure 13:**
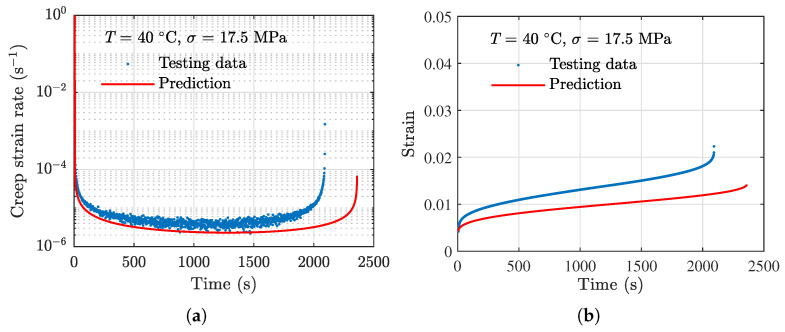
Comparison of the model prediction and the actual data of the validation specimen. (**a**) The creep strain rate vs. time, and (**b**) the creep strain vs. time.

Testing data of the validation specimen (No. 5 in Table 1) are used for validation. Using Equations (14) and (15) with the corresponding temperature and stress of the specimen, the creep strain results are obtained. Comparison between the model prediction results and the actual data are presented in Figure 13. It can be seen that the model can effectively capture the three stages of the creep process.

To further investigate the generality of the proposed model, the third-party testing data on high-density polyethylene (HDPE) are employed [55]. The testing data consist of creep strain results of a total number of seven specimens. Model parameters of Equation (5) are obtained using data associated with the seven specimens. The response surface model coefficients are subsequently obtained using Equation (10). The resulting temperature- and stress-dependent model parameters for the third-party testing data are

(17) $$\left\{\begin{array}{c} a\left(T,\sigma \right)\;=0.05975\cdot T+0.3103\cdot \sigma -9.876\\ b\left(T,\sigma \right)\;=-0.001301\cdot T-0.003743\cdot \sigma +0.04663\\ c\left(T,\sigma \right)\;=-0.001239\cdot T-0.001089\cdot \sigma +0.1145 \end{array}\right..$$

**Figure 14:**
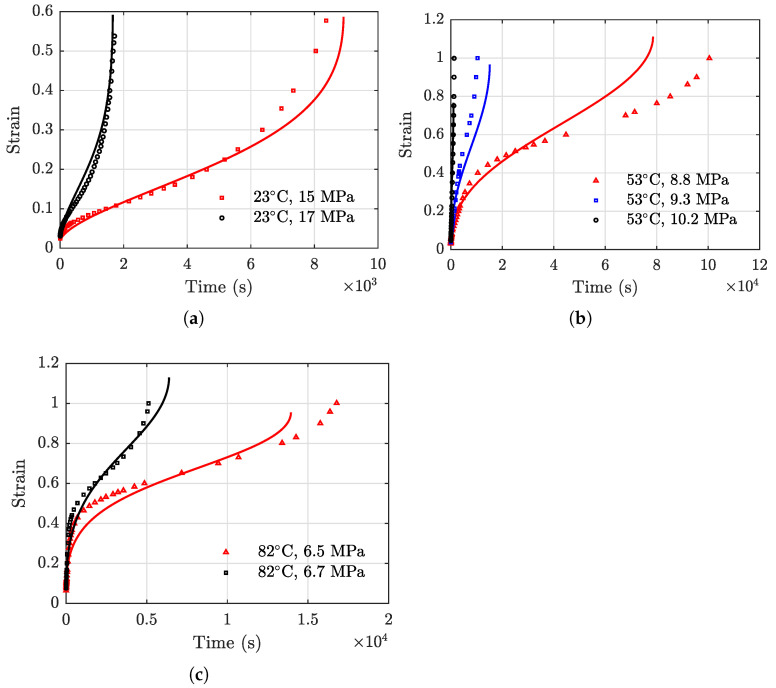
Comparisons of the model prediction results and the actual creep strain data reported in Ref. [55]. (**a**) 23 °C, (**b**) 53 °C, and (**c**) 82 °C.

Using Equation (15) and the above model parameters, the creep strain prediction results are obtained and presented in Figure 14. A general close agreement between the model prediction results and the actual testing data can be observed, indicating the proposed model can be effectively used for creep strain prediction for other PBMs with similar strain behaviors. RMSEs of the model prediction results are evaluated and shown in Table 4 where the maximum value is 0.1196 under the condition of 53 °C and 8.8 MPa.

### 4.2. Model Comparisons

To further demonstrate the performance of the proposed model, the model is compared with a reference model reported in Ref. [51]. The reference model in Equation (18) can also describe the primary–secondary–tertiary creep behavior.

(18) $$\frac{d\varepsilon }{dt}=\frac{\varepsilon P_{1}}{t}\cdot \left[\frac{1+P_{2}\left(P_{3}\varepsilon \right)+P_{4}{\left(P_{3}\varepsilon \right)}^{2}}{1+P_{1}+\left(2+P_{1}\right)P_{2}P_{3}\varepsilon +\left(3+P_{1}\right)P_{4}{\left(P_{3}\varepsilon \right)}^{2}}\right],$$

where $P_{1}$, $P_{2}$, $P_{3}$, $P_{4}$ are fitting parameters and other variables are defined as before.

**Figure 15:**
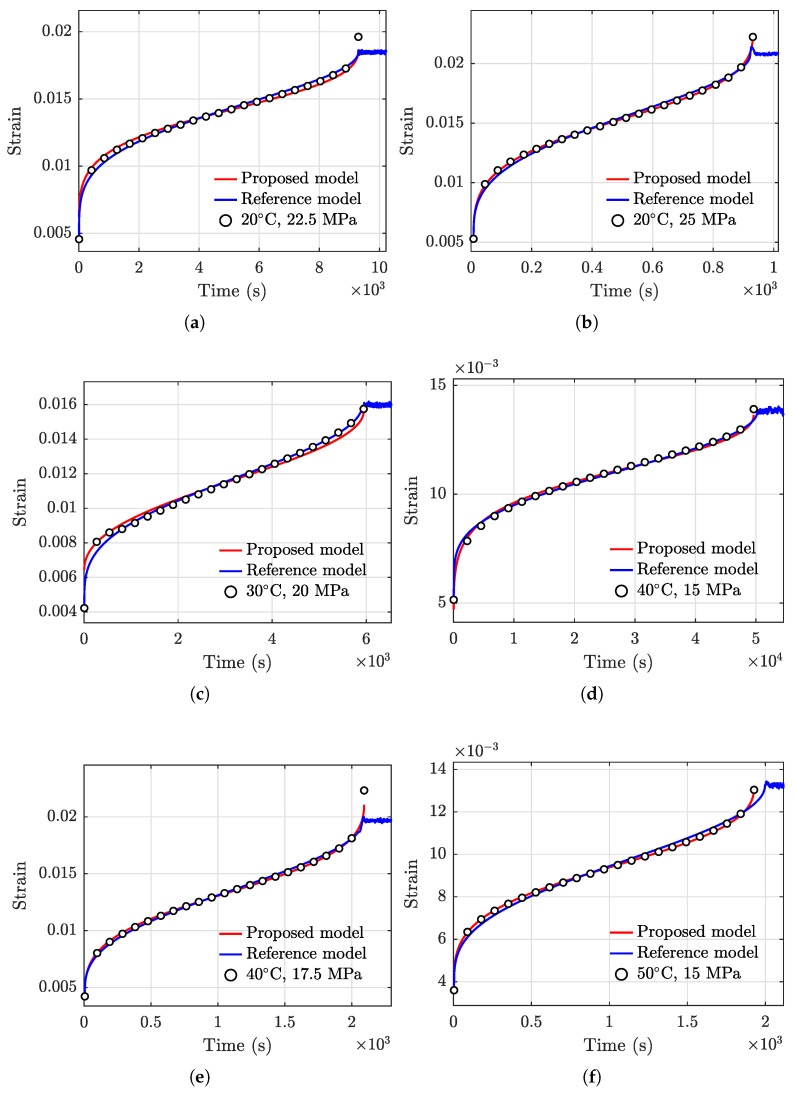
Comparisons of the results obtained using the proposed model and that using the reference model [51]. The discrete markers represent testing data. (**a**) 20 °C, 22.5 MPa, (**b**) 20 °C, 25 MPa, (**c**) 30 °C, 20 MPa, (**d**) 40 °C, 15 MPa, (**e**) 40 °C, 17.5 MPa, and (**f**) 50 °C, 15 MPa.

The same data in Table 1 for modeling are used to obtain the required model parameters of Equation (18). The prediction results of creep strain are evaluated using the proposed model and the reference model, and are compared with the actual testing data in Figure 15. In general, the two models both yield satisfactory fitting results.

**Figure 16:**
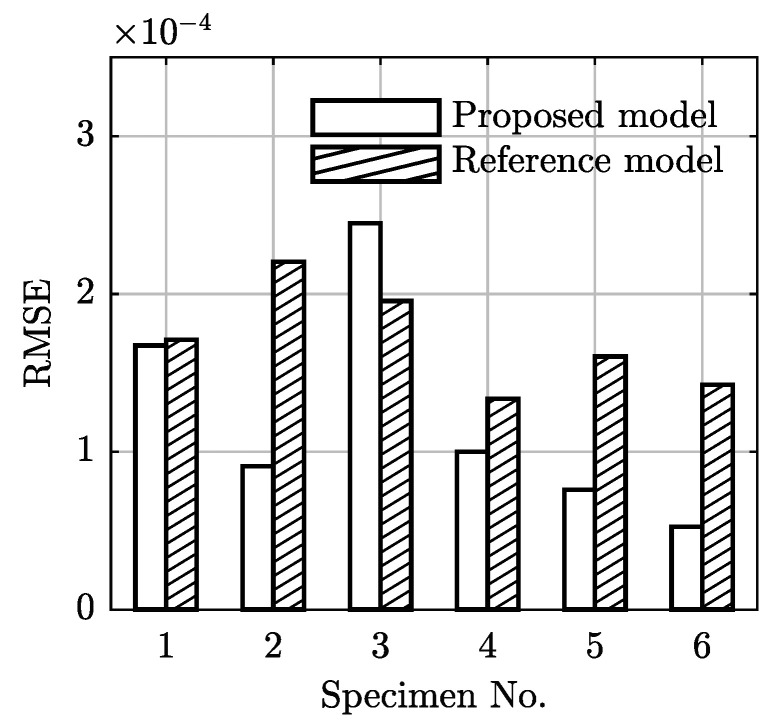
Comparisons of the performance in terms of RMSE between the proposed model and the reference model.

RMSEs of the two models under each of the conditions are evaluated and compared in Figure 16, where the proposed model yields smaller RMSEs in all data sets except for the case of (30 °C, 20 MPa). The sum of the RMSEs associated with the proposed model and the reference model are $0.73\times 10^{-3}$ and $1.02\times 10^{-3}$, respectively. The proposed model reduces the overall RMSE by about 28.5%.

In addition, the proposed model predicts the tertiary creep stage more reliably than the reference model. Fluctuations of the resulting creep strains produced by the reference model can be observed when the creep strain approaches the rupture life, as shown in Figure 16. For the strain rate equation of Equation (18), there exists a critical strain larger than which a negative strain rate can be produced by the equation. The negative strain rate reduces the creep strain to a value lower than the critical strain, leading to a positive strain rate again. This alternating nature of the polynomial function causes the strain rate varies between the negative and positive values. Consequently, the resulting strain fluctuates around the critical strain.

## 5. Conclusions

A unified phenomenological creep model was developed for polymer-bonded composite materials, allowing for predicting the creep behavior in the entire primary, secondary, and tertiary stages. A total number of six specimens made of a typical polymer-bonded composite material were prepared. The uni-axial compression creep testing with the holding stress in the range of 15 MPa–25 MPa at a temperature ranging from 20 °C to 50 °C was performed to acquired creep strain data. A rational function-based equation was proposed to describe creep strain rate in the entire creep process. The model parameters were identified using testing data. The temperature- and stress-dependent effect was incorporated into the strain rate model using a first-order response surface model. The creep rupture time model based on the Larson–Miller parameter is established, allowing for predicting the creep strain under more general conditions without testing data. The effectiveness of the model was verified using data of an independent specimen and reported creep data on another type of PBM. Furthermore, the performance of the proposed model was compared with an existing full-stage creep model. The performances of the two models in terms of RMSE were quantified and compared. Based on the current results, the following conclusions were drawn.

- The developed unified phenomenological creep model can describe the full primary–secondary–tertiary creep process under more general conditions of temperature and stress. The effectiveness of the model was validated using both independent and third-party testing data.
- The Larson–Miller parameter can be used for predicting the rupture time of PBMs. Combined with the proposed strain rate model, it can be used to predict the creep behavior under more general conditions without testing data on rupture life.
- The developed model was compared with an existing reference model. Results show that the developed model is more accurate in terms of root mean squared error. For the testing data used in this study, the proposed model reduces the overall errors by 28.5%. In addition, the proposed model is more reliable for the tertiary creep prediction due to the monotonic strain rate equation.

It is worth mentioning that the proposed model is phenomenological in nature and it cannot explain the detailed creep mechanisms of PBMs. However, it provides a viable means for creep strain and rupture time prediction using one unified model under more general conditions of temperature and stress. At least three sets of testing data on the entire creep process are required to identify the required parameters in the proposed model. 

## Figures and Tables

**Table 1:** Testing conditions and data usage.

No.	*T* (°C)	σ (MPa)	Usage
1	20	22.5	Modeling
2	20	25	Modeling
3	30	20	Modeling
4	40	15	Modeling
5	40	17.5	Validation
6	50	15	Modeling

**Table 2:** Results of model fitting parameters (a,b,c) using Equation (5). The initial and rupture times are directly obtained from the raw testing data. The numbers in the first column corresponds to the test condition in Table 1.

No.	*T* (°C)	σ (MPa)	*a*	*b*	*c*	$t_{\mathrm{ini}}$ (s)	$t_{\mathrm{rup}}$ (s)
1	20	22.5	−5.763	−0.6108	−0.04702	7.491	9299
2	20	25	−5.052	−0.07327	−0.06429	8.127	930.9
3	30	20	−7.200	−0.04438	−0.03786	6.406	5944
4	40	15	−6.334	−0.05687	−0.03744	37.00	49637
6	50	15	−5.671	−0.06509	−0.05838	4.945	1516

**Table 3:** Model performance in terms of RMSE.

*T* (°C)	σ (MPa)	RMSE
20	22.5	0.001073
20	25	0.002222
30	20	0.002700
40	15	$3.737\times 10^{-4}$
50	15	$9.743\times 10^{-4}$

**Table 4:** RMSEs of the model prediction for the creep strain data reported in Ref. [55].

*T* (°C)	σ (MPa)	RMSE
23	15	0.03264
23	17	0.03817
53	8.8	0.1196
53	9.3	0.09552
53	10.2	0.009159
82	6.5	0.07257
82	6.7	0.05441

## Data Availability

All data are included within the text.

## Abbreviations

The following abbreviations are used in this manuscript:

PBMs	Polymer-bonded composites materials
CT	Computed tomography
RMSE	Root mean square error
